# Size-dependent validation of MODIS MCD64A1 burned area over six vegetation types in boreal Eurasia: Large underestimation in croplands

**DOI:** 10.1038/s41598-017-03739-0

**Published:** 2017-07-05

**Authors:** Chunmao Zhu, Hideki Kobayashi, Yugo Kanaya, Masahiko Saito

**Affiliations:** 1 Research and Development Center for Global Change, Japan Agency for Marine-Earth Science and Technology, Yokohama, 2360001 Japan; 2 Institute of Arctic Climate and Environmental Research, Japan Agency for Marine-Earth Science and Technology, Yokohama, 2360001 Japan

## Abstract

Pollutants emitted from wildfires in boreal Eurasia can be transported to the Arctic, and their subsequent deposition could accelerate global warming. The Moderate Resolution Imaging Spectroradiometer (MODIS) MCD64A1 burned area product is the basis of fire emission products. However, uncertainties due to the “moderate resolution” (500 m) characteristic of the MODIS sensor could be introduced. Here, we present a size-dependent validation of MCD64A1 with reference to higher resolution (better than 30 m) satellite products (Landsat 7 ETM+, RapidEye, WorldView-2, and GeoEye-1) for six ecotypes over 12 regions of boreal Eurasia. We considered the 2012 boreal Eurasia burning season when severe wildfires occurred and when Arctic sea ice extent was historically low. Among the six ecotypes, we found MCD64A1 burned areas comprised only 13% of the reference products in croplands because of inadequate detection of small fires (<100 ha). Our results indicate that over all ecotypes, the actual burned area in boreal Eurasia (15,256 km^2^) could have been ~16% greater than suggested by MCD64A1 (13,187 km^2^) when applying the correction factors proposed in this study. This implies the effects of wildfire emissions in boreal Eurasia on Arctic warming could be greater than currently estimated.

## Introduction

Wildfires have an important effect on vegetation dynamics, the biogeochemical cycles of carbon, nitrogen, and other elements, atmospheric chemistry, and the climate. Globally, 301–377 Mha of land were burned annually during 1997–2011^1^. In a warmer world, the frequency, size, and number of extreme fires and the length of the fire season are predicted to increase^2–5^. Boreal Eurasia is one region where large areas of land are burned every year^6–9^. Numerous particles and large volumes of greenhouse gases are emitted by such fires^10–12^, and these pollutants are transported to East Asia^13, 14^, the western North Pacific^15, 16^, and the Arctic^17^.

Atmospheric transport of fire-emitted pollutants (e.g., black carbon particles) to the Arctic and their subsequent deposition is believed to accelerate Arctic warming^18, 19^. Forest fires in Russia provide one of the largest contributions to Arctic surface temperature change^20^. A recent simulation study indicated that 68% of black carbon deposited over the Arctic could originate from biomass burning, of which boreal Eurasian vegetation fires could contribute 85%^21^. However, the contribution of boreal fires to climate change in the Arctic is highly variable depending on the emission inventories used. Despite much development, current numerical models remain incapable of resolving the observed seasonal variations of black carbon in the Arctic, suggesting an underestimation of the black carbon source in boreal Eurasia in the emission inventory used^22, 23^.

The Global Fire Emissions Database (GFED) is a widely used fire emission inventory^1^. As its basis, the GFED adopts burned area products detected by the Moderate Resolution Imaging Spectroradiometer (MODIS) sensor, incorporating information of fuel loading, combustion completeness, and emission factors. The latest version of GFED, GFED version 4 uses the MODIS MCD64A1 burned area product^24^. The MCD64A1 product was generated based on a hybrid algorithm that incorporates active fires and changes in multitemporal spectral indices^24^.

Uncertainties could be introduced to the MCD64A1 product because of the “moderate resolution” characteristic (500 m). Burned area patches are covered by ash, charcoal, and scorched leaves and thus, they are characterized by low spectral reflectance compared with the pre-burn condition. Depending on the vegetation (fuel) type, fuel load, and soil texture, the sensor detection of pre- and post-fire imagery might introduce signals other than those that reflect the true occurrence of fire^25, 26^. Therefore, comparison of the burned areas derived from MODIS with those generated from higher resolution satellite products could provide fundamental information regarding the accuracy and future applicability of the burned area products.

Using stratified random sampling and reference data, Padilla *et al*. found that the MODIS MCD45 product could detect only 48% of the global burned area^27^. However, few studies have addressed the validation of the MCD64A1 product^28^, especially in boreal Eurasia. Recently, based on QuickBird and WorldView-2 (resolution: <5 m) reference imagery, Hall *et al*. showed that both MCD45A1 and MCD64A1 were unable to map approximately 95% of burned areas in Russian croplands, raising concerns regarding the application of these products to burned areas associated with agricultural fires^29^.

In 2012, wildfires with sizes ranging from approximately 10 to 5000 ha occurred across a wide region of boreal Eurasia, while historically low sea ice extent was recorded in the Arctic^30^. Although changes in the general circulation patterns of the atmosphere and oceans might have had significant effect in 2012, the climatic effects of pollutants could have been one of the important factors driving the dramatic change. However, the quantities of pollutant emissions from wildfires are largely variable depending on the inventories adopted. A newly developed Fire Emission Inventory–northern Eurasia (FEI-NE) has indicated that the average black carbon emission in 2002–2015 was 3.2 times higher than the GFED4^31^. Interestingly, the FEI-NE black carbon emissions in 2012 (1.17 Tg) are 4.9 times greater than GFED3 (0.24 Tg)^21^. To understand better the effects of wildfires on the Arctic climate, it is necessary to improve the estimation of the burned area.

**Figure 1:**
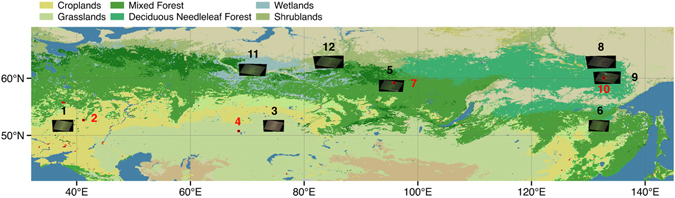
Distributions of 12 validation regions in boreal Eurasia over a wide range of vegetation types. Landsat 7 ETM+ reference images are numbered in black. Reference images from commercial satellite products are numbered in red. Only the major vegetation types studied in this work are labelled. Vegetation type data are from the MODIS MCD12Q1 product. Detailed information of the 12 regions is presented in Table S1. The figure is created using QGIS 2.14.0 (http://www.qgis.org).

This work focused on the 2012 burning season (mostly July–September) to determine correction factors for the total area burned via validation of the MODIS MCD64A1 burned area product. Products from the Landsat 7 ETM+, RapidEye, WorldView-2, and GeoEye-1 (resolution: 30, 5, 2, and 2 m, respectively) satellites were used to generate reference burned areas that allowed us to investigate the size-dependent fire occurrence patterns. We endeavoured to incorporate all cloud-free burned regions of as many vegetation types as possible over Russia in 2012. We investigated 12 regions from western Russia/Kazakhstan to eastern Siberia in which severe wildfires had occurred in croplands, grasslands, mixed forests, deciduous needleleaf forests, wetlands, and shrublands (Fig. 1). We estimated the fire polygon count detection rate (FCDR), burned area detection rate (BADR), and detection errors. Based on the ratio between the MCD64A1 and reference (MCD/Ref) burned areas, we proposed a correction factor for each region and vegetation type that should be applied to the fire emission product before its implementation in chemistry and climate models in boreal Eurasia.

## Results

### Reference fire occurrence

**Figure 2:**
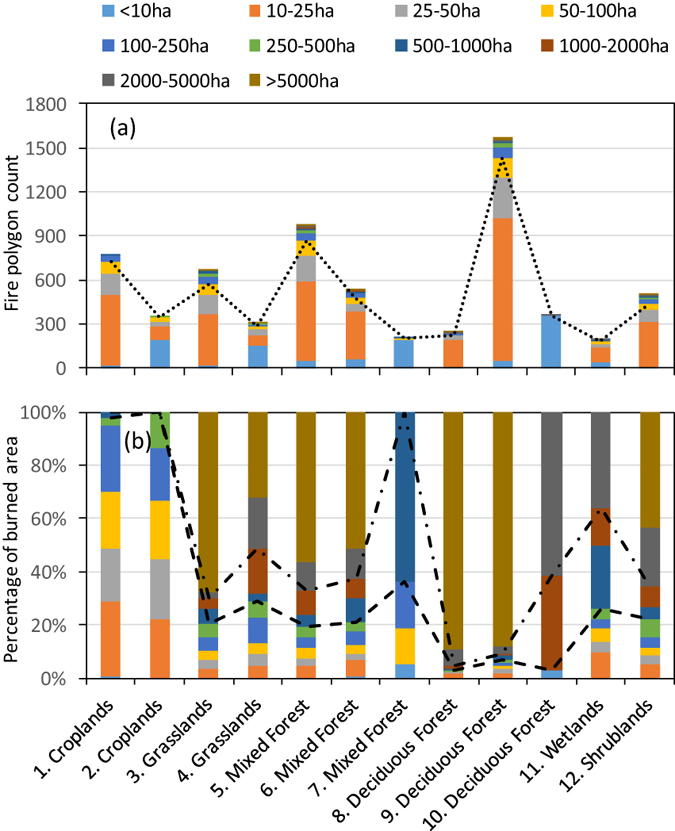
Fire occurrence in the reference satellite products as a function of fire size shown by (**a**) fire polygon count and (**b**) percentage of burned area in the region (burned area fraction). Dotted line in (**a**) indicates the boundary of fire counts for fires of 100 ha. Dashed and dashed–dotted lines in (**b**) indicate the boundaries of burned area percentage for fires of 500 and 2000 ha, respectively.

Given the high spatial resolution of the reference imagery, large numbers of small fires were detected (Table 1, Fig. 2a). Over the 12 regions, 86–99% of fire polygon counts were <100 ha in size. In croplands, 95% of the total 1123 fire polygon counts were <100 ha in size, accounting for 70% of the total burned area. Small-scale fires were the dominant cause of burned land areas in croplands. Conversely, for vegetation types other than croplands, 64–97% of burned land areas were >500 ha in size, shown as those parts above the dashed line in Fig. 2b. Specifically, 92%, 66%, and 67% of the total burned areas in deciduous needleleaf forests, mixed forests, and grasslands, respectively, were >2000 ha. In this study, we considered fires with burned areas >2000 ha as megafires. Similarly, 74% and 78% of the total burned areas in wetlands and shrublands, respectively, were >500 ha. These results indicate that for vegetation types other than croplands in boreal Eurasia, although large numbers of small fires were detected, the dominant land burns were caused by large-scale fires.

**Table 1:** General results of validation of MCD64A1 burned area products in boreal Eurasia.

id	Biome	Fire polygon count			Burned area (ha)			Fire detection rates		MCD/Ref ^b^	Correction Factor
		Reference	MCD64A1	Overlapped	Reference	MCD64A1	Overlapped	Count (%)	Area (%)^a^		
1	Croplands	770	18	3	26,522	3221	86	0.4	0.3	0.12	8.2
2	Croplands	353	10	6	6383	1075	210	1.7	3.3	0.17	5.9
3	Grasslands	666	12	6	149,900	140,808	86,386	0.9	58	0.94	1.1
4	Grasslands	308	24	14	26,052	37,516	11,468	4.5	44	1.44	0.69
5	Mixed forests	978	141	102	199,006	196,761	96,543	10	49	0.99	1.0
6	Mixed forests	533	63	32	80,430	148,311	52,504	6.0	65	1.84	0.54
7	Mixed forests	199	8	4	1428	6303	1227	2.0	86	4.42	0.23
8	Deciduous needleleaf forests	244	8	7	176,961	180,283	134,624	2.9	76	1.02	0.98
9	Deciduous needleleaf forests	1570	37	25	746,934	469,409	378,367	1.6	51	0.63	1.6
10	Deciduous needleleaf forests	354	1	1	3843	1408	1305	0.3	34	0.37	2.7
11	Wetlands	194	100	32	17,876	38,147	9879	17	55	2.13	0.47
12	Shrublands	500	140	70	90,307	95,439	27,051	14	30	1.06	0.95

^a^Area (%) indicates the burned area detection rate (BADR) of MCD64A1.

^b^MCD/Ref indicates the ratio between MCD64A1 and reference burned area.

### Fire detection capability of MCD64A1

**Figure 3:**
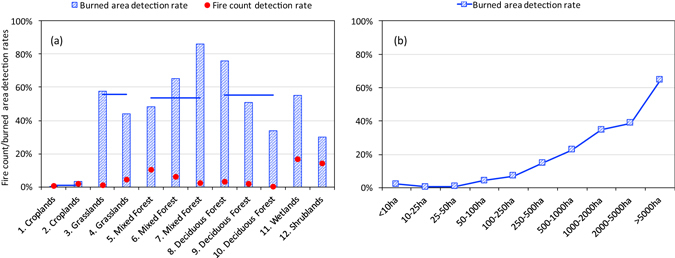
Fire count detection rate and burned area detection rate of MCD64A1 in boreal Eurasia shown as a function of (**a**) vegetation type and (**b**) fire size (only for burned area detection rate). Horizontal bar in (**a**) indicates the average burned area detection rate of the corresponding vegetation type.

The fire detection capability of the MCD64A1 product was evaluated quantitatively based on the FCDR and BADR (Fig. 3). For the different regions, the FCDR was within the range 0.3–17.0%. A large number of small fires (<100 ha) was not detected well by MCD64A1. In croplands, the FCDR and BADR were within the range 0.4–1.7% (simultaneous estimations on two cropland regions of 0.8%, same as follows) and 0.3–3.3% (0.9%), respectively. For vegetation types other than croplands, the BADR was within the range 30–86%. Specifically, the BADR was within the range 34–76% (55%) in deciduous needleleaf forests, 49–86% (54%) in mixed forests, and 44–58% (56%) in grasslands. In wetlands and shrublands, the BADRs were 55 and 30%, respectively. Overall, in comparison with the reference images, MCD64A1 underestimated the burned areas.

As a function of fire size, the BADR increased from <10% for areas <100 ha to a maximum of 65% for areas >5000 ha (Fig. 3b). However, even for megafires, MCD64A1 could reproduce only ~2/3 of the true land burns. Such a result suggests the burned area products and the GFED4 emission inventories could be improved for boreal Eurasia before their application in chemistry and climate models.

### Detection errors

The disagreement between the MCD64A1 and reference burned areas was quantified based on commission errors and omission errors, which were further treated as independent and related errors, respectively. Independent commission errors (ICEs) were generally low in boreal Eurasia (Fig. S1a). Mixed and deciduous forests showed the lowest ICEs of 0.01–1.60%, indicating that almost all fires detected by MODIS were also detected by Landsat 7 ETM+ in these forest types. Conversely, relatively high ICEs were detected in croplands (20–44%), and a relatively high ICE level (16%) was estimated for wetlands.

In comparison with ICEs, the independent omission errors (IOEs) showed higher values (Fig. S1a). In forests, except for region id 9 (Fig. 1, Table S1), the IOEs were within the range 3.5–39.0%. In region id 9, which is covered by deciduous forests, MODIS detected only two of 1431 fires of <100 ha and only 25 of the total 1570 fires, leading to a high IOE value of 99.5%. High IOE values were also estimated for croplands (90–99%). However, both ICEs and IOEs showed decreasing trends as the fire size increased (Fig. S1b). Larger fires, which often persist longer, tend to produce clear differences in spectral reflectance that are detected more easily by satellite.

The values of related commission errors (RCEs) ranged from 17% on average (range: 7.3–25.0%) in deciduous forests to 85% (76–95%) in croplands, 36% (33–38%) in grasslands, 65% (50–80%) in mixed forests, 69% in wetlands, and 72% in shrublands (Fig. S2a). Like RCEs, relatively high values of related omission errors (ROEs) were estimated for croplands (66%; range: 64–69%). The RCEs and ROEs did not show clear patterns among the different vegetation types but they did decrease markedly as fire size increased (Fig. S2b).

### MCD64A1/reference burned area ratios and correction factors

The MCD64A1 burned area could be corrected for further regional-scale applications. MCD/Ref ranged from 0.13 (0.12 and 0.17 for regions id 1 and id 2, respectively) in croplands to 2.13 in wetlands (Table 1). Relatively close MCD/Ref values were estimated for grasslands (1.01), shrublands (1.06), mixed forests (1.25), and deciduous needleleaf forests (0.70). Corresponding to the MCD/Ref in each vegetation type, we suggest multiplying the MCD64A1 product burned areas for boreal Eurasia by correction factors of 0.23–8.20, depending on the region, prior to their application to emission estimations (Table 1). On an ecotype basis, the correction factors were estimated as 7.70 for croplands, 0.80 for mixed forests, 1.40 for deciduous forests, 0.47 for wetlands, 0.99 for grasslands, and 0.95 for shrublands.

## Discussion

**Figure 4:**
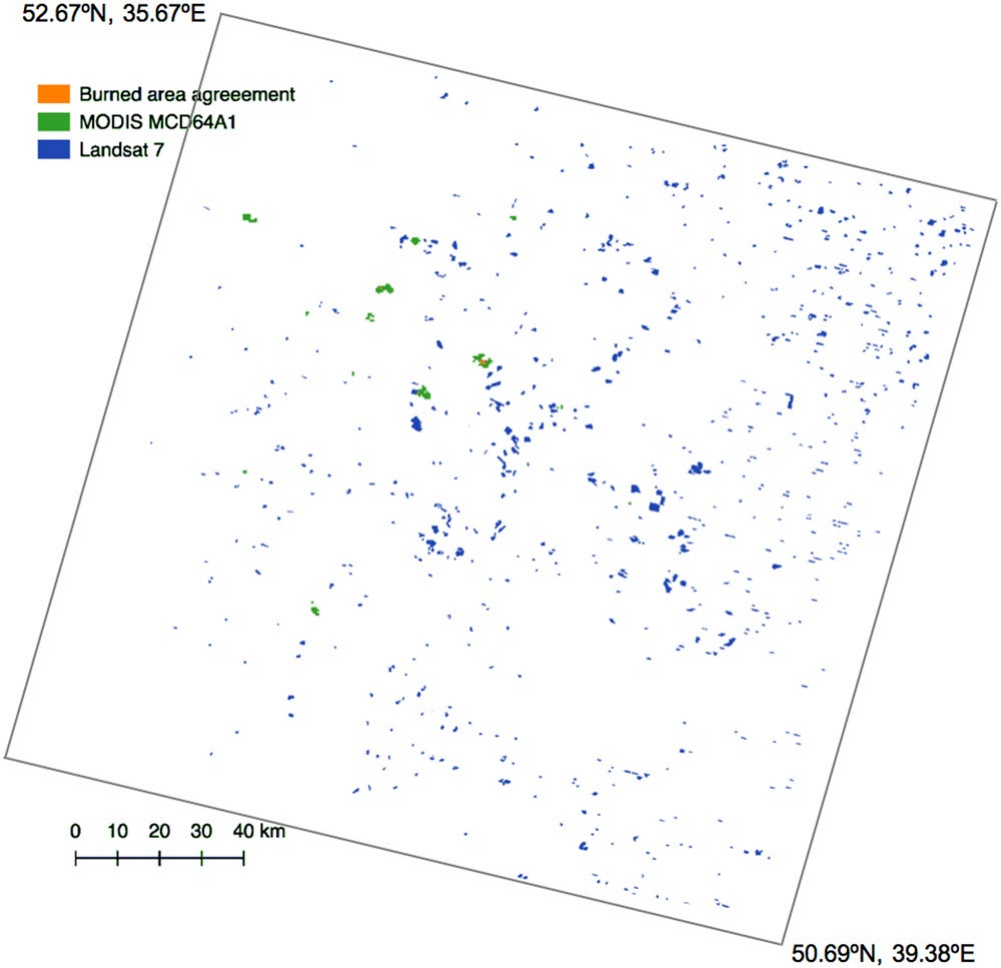
Burned area map showing a comparison of MCD64A1 and Landsat 7 ETM+ products for croplands in region id 1 in Fig. 1.

In croplands, only a very small fraction of burned regions in the reference satellite products was detected by MCD64A1 (Fig. 4). Based on direct comparison of pre- and post-fire imagery, our results indicate that MCD64A1 could detect more of the reference burned area (13%) in croplands than estimated by Hall *et al*. (~5%) who used an opportunistic acquisition strategy to sample the reference imagery^29^. The low detectability of MCD64A1 is related to the coarse spatial resolution (500 m), which means small fires of <120 ha in size (most cropland fires were <100 ha) are not detected well^24^. Fires in croplands are mainly caused by human activities such as maintenance burning for the purposes of clearing residues prior to planting or post harvesting^25, 32^. Cropland fires, therefore, are characterized by small sizes, transient nature, and non-contiguous patches^33^. Our results on fire size distribution in boreal Eurasia are implicative to the implementation of fire-control policies.

The BADR results in this study are smaller than the global assessment of accuracy of MCD45A1 in which 84% of Landsat 7 ETM+ burned areas were estimated correctly in central Siberia^24^. Oliva and Schroeder estimated a higher BADR of 86% using the VIIRS 375 m active fire detection product and Landsat 8 reference imagery in eastern Siberia^34^. The BADR differences between our results and previous studies might be caused by differences in the thresholds used for estimating the difference of the normalized burn ratio (dNBR) between the post- and pre-fire reference imagery. Focusing on Russia, we determined the dNBR threshold based on each image, which we considered would suggest estimates of burned areas that are more realistic. The selection of reference imagery products might also contribute to the BADR differences. In addition to Landsat 7 ETM+, we also used higher resolution reference burned area data from RapidEye, GeoEye-1, and WorldView-2 imagery in the validation of the MCD64A1 burned area product.

Low ICEs in mixed and deciduous forests might be related to the plentiful supply of wood fuel in these forests, relatively high flame temperature, and long burning duration, all factors that favour detection by satellite sensors via reflectance changes^24, 28^. Interestingly, only 52 of 66 megafires >2000 ha were detected by the MODIS sensor, causing an increase in IOEs (Fig. S1b). MODIS missed five megafires in mixed forests, five in deciduous forests, one in grasslands, and three in shrublands. In forests and shrublands, it is possible that fires could persist predominantly within the ground when there is plenty of fuel on the floor, and if not in a time of drought, evidence of such fire at canopy level might be lacking. Further studies on the relations of fire occurrence with regional vegetation dynamics are needed. The masking effect of cloud cover, although was removed in the reference imagery during data processing, could influence the MCD64A1 burned area by interfering with the spectral reflectance, leading to increased omission errors and underestimation^1^.

In wetlands, the high ICE value might incorporate interference from water bodies, which are characterized by very low spectral reflectance. Further, a high RCE value (69%) was incorporated in the BADR (55%), which caused considerable overestimation of the burned area by MCD64A1 in wetlands. In croplands, surface spectral reflectance is not only reduced by burning but also by ploughing practices, residues, and even dark soils, which could be misconstrued as burned land, resulting in high ICEs. Mollisol soil in particular is black in appearance and it has very low spectral reflectance (<0.1) in the 350–2500 nm range^29^. As Mollisol and similar black soil types, e.g., Vertisol and Chernozem, account for 53% of arable lands in the Russian Federation^29, 35^, detection errors of MODIS burned area in croplands might be introduced by taking the reflectance of black soil. Our results on the high IOE values in croplands are in accordance with Hall *et al*. who reported similar omission errors of MCD64A1 (99.2–96.7%)^29^.

The differences in the resolutions of MODIS and the reference satellites could contribute to RCEs and ROEs. One MODIS pixel of 25 ha encompasses ~278 Landsat 7 ETM+ pixels and even more for the very high resolution satellite products. Thus, for MODIS products, unburned regions within a fire perimeter might be recognized as burned, while some burned parts at the boundary of a fire perimeter might not be distinguished, resulting in increases of RCE and ROE, respectively. In croplands, RCEs could also be attributed to land use and soil texture, as with the ICEs. Moreover, characterized by short duration at relatively low flame temperatures, a narrow flaming front, and a short smouldering stage^36^, maintenance fires in croplands intended to clear residues might not be detected by MODIS MCD64A1, leading to an increase of ROEs.

In croplands, we used a sampling interval of <20 days for pre- and post-fire reference imagery selection. Although it might cause interference signals from agricultural activities to burned area estimation, to our knowledge, this is the best attempt of this kind of study so far. Recent studies have highlighted the importance of cropland fires in boreal regions with regard to their large emissions of black carbon and its subsequent transport to the Arctic^37, 38^. Therefore, accurate assessment of burned areas in croplands is required urgently, which will necessitate the deployment of satellites/sensors with high spatial resolution and high revisit frequency, in conjunction with the improvement of burned area estimation algorithms.

Based on the correction factors, of the 12 studied regions over the wide extent of boreal Eurasia in 2012, the burned area was corrected from 13,187 km^2^ (detected by MCD64A1) to 15,256 km^2^ (detected by the reference satellites). These results suggest that emissions of short-lived climate pollutants such as black carbon and CO from biomass burning in Eurasia could be ~16% higher than current estimates. This value is attributed merely to burned area underestimation without consideration of vegetation-specific emission factor variations or combustion stage differences. These correction factors could be used directly in chemistry and climate models corresponding to each region in boreal Eurasia (Table 1). The FEI-NE indicated that forests constitute a large source of black carbon because of substantial fuel loading^31^. Over the entire region of forest investigated in this study, the burned area could be ~20% higher than current estimates. As the burned area data of FEI-NE are based on the MODIS products, the actual black carbon emissions from forest in 2012 could have been 1.2 times of current estimates. Such results suggest that the effects of wildfires in boreal Eurasia on rapid warming of the Arctic might be greater than currently believed.

It should be noted that the sensitivity of the detected burned areas to the band thresholds of the estimation algorithms might also contribute to the nominal accuracy level. In comparison with the probabilistically based determination of algorithm thresholds of MCD64A1^24^, our attempts to determine the burned regions by visual comparison of the pre- and post-fire satellite images and subsequent tuning of the thresholds on a region-by-region basis suggest burned area results closer to reality. Future studies addressing onsite validation of remote sensing burned area estimation, such as by field investigation, would provide further information that is more accurate about the uncertainties of both the MCD64A1 and higher resolution satellite products. Nevertheless, our results are essential for better understanding of both the biosphere–atmosphere exchange of trace gases and particles and the fire–climate relationship.

## Methods

### Validation procedures

Our rationale was to select reference imagery, calculate the burned areas, and compare them with the MCD64A1 product. We first examined the fire occurrence pattern in boreal Eurasia based on MCD64A1. We found that southwestern Russia, Kazakhstan, southwest Siberia, central Siberia, eastern Siberia and the Far East are the major fire-prone regions (Fig. S3). Pairs of reference satellite products, i.e., pre- and post-fire imagery, were then selected mainly in these regions. Recently, Boschetti *et al*. proposed a stratified random sampling method which could generate statistically sound samples for validation^39^. Using a different method, we believe that the screened reference imagery in this study are representative of the cases in boreal Eurasia in 2012. Similar validation strategy was also used by other studies^8, 28^.

The selection of reference imagery was based on the following criteria: (1) there were visibly notable fire scars (burned areas) in the MCD64A1 product in the region; (2) there were explicitly visible differences between the pre-fire (i.e., shown by green) and post-fire true colour imagery (i.e., shown by brown or black) in the burned areas; (3) cloud cover in the reference imagery was <10% and the visible burned places based on the true colour imagery were free of cloud; and (4) the duration between the paired pre- and post-fire imagery was <20, <60, and <100 days for croplands, grasslands, and forests, respectively. This practice was intended to retain the charcoal signal while minimizing the effect of vegetation recovery shading the evidence of fire occurrence. In grasslands where pre- and post-fire differences of true colour imagery were close, imagery where large area was burned in MCD64A1 were selected.

Burned areas in each region were then calculated using the algorithms following Oliva and Schroeder^34^. The thresholds were optimized according to the reflectance spectra distributions and differences of true colour imagery. The accuracy of the MCD64A1 product was then evaluated by assessment of the level of agreement with the burned areas of the reference products.

### Study area

Twelve pairs of satellite products distributed from southwestern Russia to eastern Siberia within the region 50–65°N, 30–140°E were selected (Fig. 1). These products included eight pairs of Landsat 7 ETM+ imagery, two pairs of RapidEye imagery, one pair of GeoEye-1 imagery, and one pair of WorldView-2 imagery. The dominant vegetation type in the study area was determined with reference to the MODIS MCD12Q1 product (http://glcf.umd.edu/data/lc/, accessed May 31, 2016). These regions covered the major fire-prone vegetation types (Table S1): (1) croplands in southwestern Russia; (2) grasslands in Kazakhstan; (3) wetlands in western Siberia; (4) shrublands in western Siberia; (5) mixed forests in central Siberia and southeastern Russia; and (6) deciduous needleleaf forests in eastern Siberia.

### MODIS MCD64A1 and reference datasets

The monthly MODIS MCD64A1 burned area product (Collection 5.1) with spatial resolution of 500 m was obtained from the University of Maryland, USA (ftp://fuoco.geog.umd.edu/db/MCD64A1/, accessed June 8, 2016). The Landsat 7 ETM+ surface reflectance reference imagery was obtained from the United States Geological Survey (http://earthexplorer.usgs.gov). The Landsat 7 ETM+ surface reflectance products have spatial resolution of 30 m and a revisit cycle of 16 days. Each Landsat 7 ETM+ image covers approximately 31,11 km^2^ (170 km N–S, 183 km E–W). The RapidEye products have spatial resolution of 6.5 m and they were resampled to 5 m. The two pairs of RapidEye imagery cover approximately 500 km^2^ (id 2; croplands) and 1032 km^2^ (id 4; grasslands) (Table S1). The WorldView-2 and the GeoEye-1 products have spatial resolution of 2 m. We selected a region of approximately 113 km^2^ in eastern Siberia where a pair of WorldView-2 images showed wildfire occurrence within 90 days (id 10; deciduous needleleaf forests). A pair of GeoEye-1 images covering a region of central Siberia of approximately 125 km^2^ was also selected (id 7; mixed forests). These commercial products were obtained through the effort of the Space Engineering Development Co., Ltd., Japan.

### Burned area mapping

The monthly MCD64A1 product contains five data layers: burn date, burn date uncertainty, quality assurance, first day of burning, and last day of burning. Corresponding to each of the reference regions, the MCD64A1 burn date data were mapped in the corresponding period. If multiple pixels were connected, they were considered one fire scar (polygon). The burned area imagery was then converted from sinusoidal projection to UTM zones 37–53 for agreement with the reference imagery.

For each pair of Landsat 7 ETM+ reference images, the inherent water mask layers of Landsat 7 ETM+ products for both pre- and post-fire images were applied. Thresholds were also applied to the near-infrared band (NIR, B4 in Landsat 7 ETM+) to remove the interference generated by water bodies (Table S2). The burned area was then estimated based on the following algorithm:

1 $${(\text{NIR}}_{{\rm{post}}} < {{\rm{NIR}}}_{{\rm{threshold}}})\,{\rm{AND}}\,(({\rm{dNIR}} > {{\rm{dNIR}}}_{{\rm{threshold}}})\,{\rm{OR}}\,({\rm{dNBR}} < {{\rm{dNBR}}}_{{\rm{threshold}}}))\cdots $$

where NIR_post_ is the surface reflectance of post-fire imagery and dNIR is the reflectance difference in the NIR band between the post- and pre-fire imagery (B4_post-fire_ − B4_pre-fire_)^34^.

For the commercial satellite products, the radiance information was first converted to reflectance according to the time of satellite passage. Furthermore, because the commercial satellite products had no shortwave infrared band information, the difference in the normalized differential vegetation index (dNDVI) between the post- and pre-fire imagery (NDVI_post-fire_ − NDVI_pre-fire_) was used instead of dNBR. The burned area was then estimated using the following algorithm:

2 $$({{\rm{NIR}}}_{{\rm{post}}} < {{\rm{NIR}}}_{{\rm{threshold}}})\,{\rm{AND}}\,(({\rm{dNIR}} > {{\rm{dNIR}}}_{{\rm{threshold}}})\,{\rm{OR}}\,({\rm{dNDVI}} < {{\rm{dNDVI}}}_{{\rm{threshold}}}))\ldots $$

To identify burned areas, true color post- and pre-fire satellite images were inspected visually and the thresholds were tuned and optimized as appropriate (Table S2).

Because of a Scan Line Corrector (SLC) failure, parts of the Landsat 7 ETM+ imagery were masked by gap lines. Consequently, when estimating burned areas, if the fire scar (polygon) was affected by the SLC failure, it was corrected manually. This was done by filling the SLC region as burned if within a fire scar. The fire scar size was then determined using the area function of the QGIS (version 2.14.0) software.

Given the 500-m spatial resolution of MCD64A1, the minimum detectable size of a burned area (one pixel) would be 25 ha. For the reference satellites, the nominal minimum sizes of burned areas were 0.09 ha for Landsat 7 ETM+, 0.0025 ha for RapidEye, and 0.0004 ha for both WorldView-2 and GeoEye-1. However, visualization of fires at such finer sizes would increase detection errors arising from (1) the spectral surface reflectance from satellite contains some noises due to the radiometric uncertainty of the sensor, (2) spectral reflectance and fire indices are influenced by the surface topography and water bodies, and (3) human activities such as forest clear cut could cause an erroneous-detection of burned area. Therefore, this study applied a sieve-screening procedure on the reference imagery to retain only fire scars ≥9.9 ha.

### Validation indices

The detection capability of MCD64A1 was evaluated based on the following aspects: FCDR and BADR, detection agreement and error, and MCD/Ref. An example schematic using Landsat 7 ETM+ as the reference imagery is shown in Fig. S4. The FCDR is defined as the percentage of reference fire polygon counts detected by MCD64A1. If any part of one reference fire polygon overlapped with the MCD64A1 burned area mapping, it was counted as detectable. The BADR is defined as the percentage of reference burned area detected by MCD64A1 (Eq. 3):

3 $${\rm{BADR}}={\rm{O}}/({\rm{B}}+{\rm{R}}+{\rm{O}}),\ldots $$

where R and B denote the independent and related parts of the reference burned area with respect to MCD 64A1 in one fire polygon (shown by red and blue areas in Fig. S4c, respectively), and O denotes the burned area agreement between the reference image and MCD64A1 (shown by orange Fig. S4c).

The detection errors were assessed based on commission errors and omission errors^26, 34, 40^. The commission errors indicate fires included by MCD64A1 that did not exist in the reference products, denoting false alarms and overestimation by MCD64A1. The omission errors indicate those reference fires that could not be detected by MCD64A1, indicating underestimation by MCD64A1. Furthermore, these errors could be divided into independent (ICE and IOE) and related errors (RCE and ROE), depending on whether part of the MCD64A1 burned area polygon overlapped that in the reference data. The ICE, IOE, RCE and ROE indices can be calculated as follows:

4 $${\rm{ICE}}={\rm{P}}/({\rm{G}}+{\rm{P}}+{\rm{O}}),\ldots $$

5 $${\rm{IOE}}={\rm{R}}/({\rm{B}}+{\rm{R}}+{\rm{O}}),\ldots $$

6 $${\rm{RCE}}={\rm{G}}/({\rm{G}}+{\rm{O}}),\ldots $$

7 $${\rm{ROE}}={\rm{B}}/({\rm{B}}+{\rm{O}}),\ldots $$

where P and G denote the independent and related parts of the MCD64A1 burned area (shown by pink and green areas in Fig. S4c, respectively).

To provide suggestions regarding the use of MCD64A1 burned area and GFED4 in boreal Eurasia, we calculated the ratio between the total MCD64A1 burned area and that of the reference imagery (MCD/Ref) in each region/vegetation type. Appropriate correction factors were then calculated as the reciprocal of MCD/Ref.

### Data Availability

The reference burned area mappings are available upon request to the authors.

## Electronic supplementary material

Supplementary information
