# Supplementary material for: Size-dependent validation of MODIS MCD64A1 burned area over six vegetation types in boreal Eurasia: Large underestimation in croplands
Source: Sci Rep. 2017 Jul 5;7:4181. doi: 10.1038/s41598-017-03739-0 (PMC5498636; doi:10.1038/s41598-017-03739-0)
Supplement: Supplementary file 1 — Supplementary information [file 41598_2017_3739_MOESM1_ESM.pdf]

Supplementary information

Size-dependent validation of MODIS MCD64A1 burned area over six vegetation types in boreal Eurasia: Large underestimation in croplands

Chunmao Zhu*, Hideki Kobayashi, Yugo Kanaya, Masahiko Saito

Department of Environmental Geochemical Cycle Research, Japan Agency for Marine-Earth Science and Technology, Yokohama 2360001, Japan

* Corresponding author:

Email: chmzhu@jamstec.go.jp

Tel.: +81- 45-778-5365

Fax: +81- 45-778-5706

This file contains 4 figures (Figs. S1–S4) and 2 tables (Tables S1 and S2).

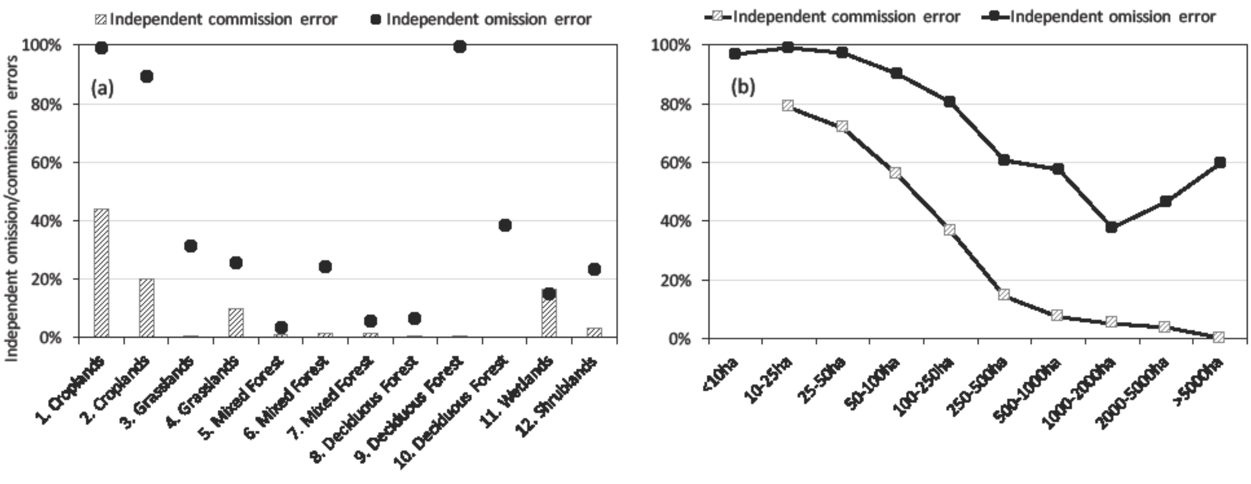

Fig. S1. Independent commission errors and independent omission errors of MCD64A1 in boreal Eurasia shown as a function of (a) vegetation type and (b) fire size.

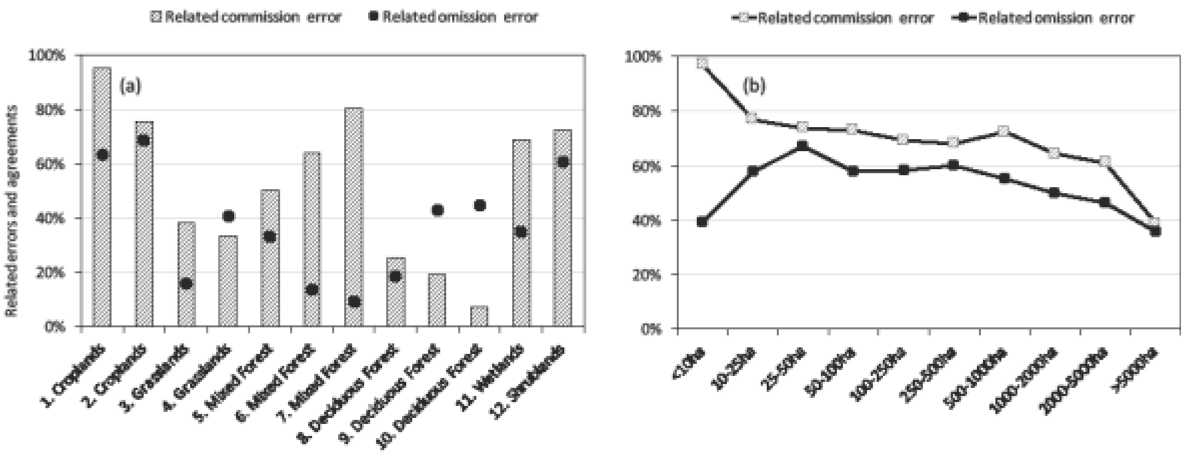

Fig. S2. Related commission errors and related omission errors of MCD64A1 in boreal Eurasia shown as a function of (a) vegetation type and (b) fire size.

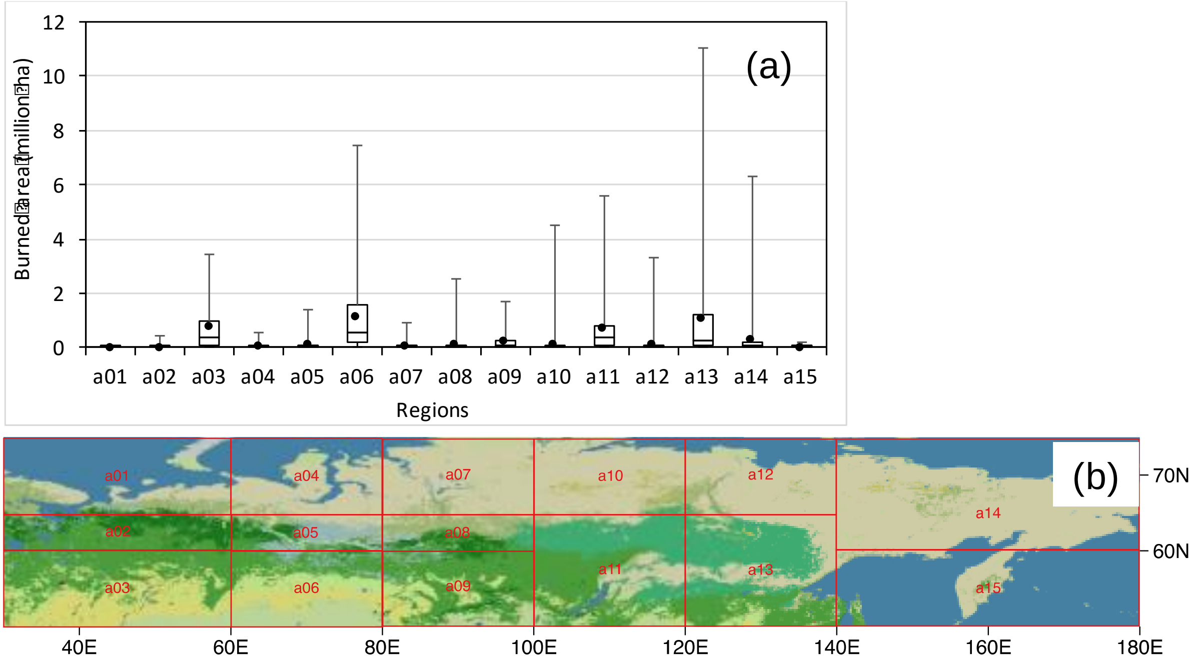

Figure S3. (a) Spatial variations of burned area pattern in boreal Eurasia in 2005–2014 based on MCD64A1. The minimum, 25th percentile, 50th percentile, 75th percentile and maximum of the burned area were shown in the box-and-whisker plot. The means were shown in solid circles. (b) Codes of regions in boreal Eurasia. The figure is created using QGIS 2.14.0 (http://www.qgis.org).

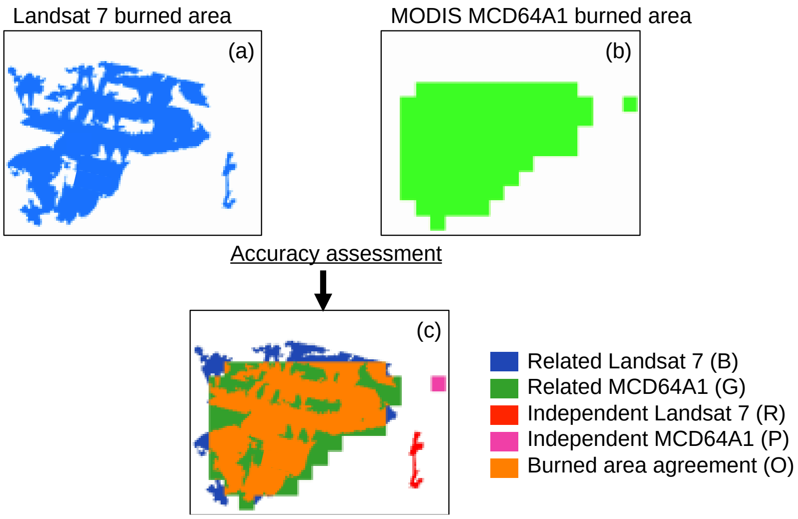

Fig. S4. Example schematic showing accuracy assessment of fire perimeters using Landsat 7 ETM+ as the reference image. The parts of Landsat 7 ETM+ in blue and red in (c) were used to compute the related omission error and the independent omission error, respectively. The parts of MCD64A1 in green and pink in (c) were used to compute the related commission error and the independent commission error, respectively. See Methods section for their definitions. Fire perimeters are taken from region id 5 in Fig. 1.

Table S1. Information of validation imagery for vegetation type, reference satellites, pre- and post-fire dates, and MODIS tiles

id	Vegetation	Region	Reference sensor	Resolution (m)	Size (km^2)*	Path/Row	Pre-fire date	Post-fire date	Duration	MODIS tiles
1	Croplands	Southwestern Russia	Landsat 7 ETM+	30	31,110	177/24	7/14/12	7/30/12	16	h20v03
2	Croplands	Southwestern Russia	RapidEye	5	500	-	7/10/12	7/30/12	20	h20v03
3	Grasslands	Kazakhstan	Landsat 7 ETM+	30	31,110	153/24	6/20/12	8/7/12	48	h22v03
4	Grasslands	Kazakhstan	RapidEye	5	1032	-	5/19/12	7/14/12	56	h22v03
5	Mixed forests	Central Siberia	Landsat 7 ETM+	30	31,110	142/19	6/7/12	8/26/12	80	h22v03, h23v03
6	Mixed forests	Southeastern Russia	Landsat 7 ETM+	30	31,110	116/24	6/1/12	8/20/12	80	h25v03, h26v03
7	Mixed forests	Central Siberia	GeoEye-1	2	125	-	7/23/12	8/25/12	33	h22v03
8	Deciduous needleleaf forests	Eastern Siberia	Landsat 7 ETM+	30	31,110	120/16	6/13/12	9/17/12	96	h23v02, h24v02
9	Deciduous needleleaf forests	Eastern Siberia	Landsat 7 ETM+	30	31,110	118/18	5/30/12	9/3/12	96	h24v02, h24v03
10	Deciduous needleleaf forests	Eastern Siberia	WorldView-2	2	113	-	6/4/12	9/2/12	90	h24v02, h24v03
11	Wetlands	Western Siberia	Landsat 7 ETM+	30	31,110	159/17	6/30/12	7/16/12	16	h21v02
12	Shrublands	Western Siberia	Landsat 7 ETM+	30	31,110	151/16	6/6/12	8/25/12	80	h21v02, h22v02

* The sizes of Landsat 7 ETM+ imagery are approximate estimates based on the length of 170 km north-south by 183 km east-west.

Table S2. Thresholds used to calculate burned areas for Landsat 7 ETM+ and commercial satellite imagery

id	Biome	Reference sensor	Water mask	NIR post <	dNIR >	dNBR <	dNDVI <
1	Croplands	Landsat 7 ETM+	0.050	0.10	0.05	0.2	-
2	Croplands	RapidEye	0.050	0.10	0.05	-	0.400
3	Grasslands	Landsat 7 ETM+	0.030	0.15	0.01	0.2	-
4	Grasslands	RapidEye	0.050	0.15	0.01	-	0.120
5	Mixed forests	Landsat 7 ETM+	0.030	0.12	0.05	0.2	-
6	Mixed forests	Landsat 7 ETM+	0.030	0.15	0.01	0.2	-
7	Mixed forests	GeoEye-1	0.030	0.12	0.05	-	0.100
8	Deciduous needleleaf forests	Landsat 7 ETM+	0.030	0.10	0.05	0.2	-
9	Deciduous needleleaf forests	Landsat 7 ETM+	0.030	0.14	0.03	0.2	-
10	Deciduous needleleaf forests	WorldView-2	0.002	0.02	0.03	-	0.286
11	Wetlands	Landsat 7 ETM+	0.030	0.10	0.05	0.2	-
12	Shrublands	Landsat 7 ETM+	0.030	0.10	0.05	0.2	-
